# Dance/movement therapy for improving metabolic parameters in long-term veterans with schizophrenia

**DOI:** 10.1038/s41537-024-00435-7

**Published:** 2024-02-22

**Authors:** Zhaoxia Zhou, Hengyong Guan, Meihong Xiu, Fengchun Wu

**Affiliations:** 1 Hebei Province Veterans Hospital, Hebei, China; 2 Peking University HuiLongGuan Clinical Medical School, Beijing HuiLongGuan Hospital, Beijing, China; 3 Department of Psychiatry, the Affiliated Brain Hospital of Guangzhou Medical University, Guangzhou, China; 4 Guangdong Engineering Technology Research Center for Translational Medicine of Mental Disorders, Guangzhou, China; 5 Department of Biomedical Engineering, Guangzhou Medical University, Guangzhou, China

**Keywords:** Schizophrenia, Psychiatric disorders

## Abstract

Accumulating evidence has supported the implementation of dance/movement therapy (DMT) as a promising intervention for patients with schizophrenia (SCZ). However, its effect on body weight and metabolic profile in SCZ remains unclear. This study aimed to evaluate the outcome of a 12-week DMT session on weight and lipid profile in patients with SCZ using a randomized, single-blinded, controlled trial design. This study encompassed two groups of long-term hospitalized patients with SCZ, who were randomly assigned to the DMT intervention (*n* = 30) or the treatment as usual (TAU) group (*n* = 30). Metabolic markers, including weight, body mass index (BMI), fasting glucose, triglycerides, and total cholesterol were measured in both groups at two measurement points (at baseline and the end of the 12-week treatment). We found that DMT intervention significantly decreased body weight (F = 5.5, *p* = 0.02) and BMI (F = 5.7, *p* = 0.02) as compared to the TAU group. However, no significance was observed in other metabolic markers, including fasting glucose, triglycerides, and total cholesterol after treatment (all *p* > 0.05). Our study indicates that a 12-week, 24-session DMT program may be effective in decreasing body weight and BMI in long-term hospitalized patients with SCZ. DMT intervention may be a promising treatment strategy for long-term inpatients in the psychiatric department.

## Introduction

Atypical antipsychotics are commonly used to reduce and control psychiatric symptoms of schizophrenia (SCZ)^1,2^. However, they are often associated with metabolic side effects, such as weight gain, glucose dysregulation, and altered cholesterol and triglyceride metabolism^3–5^, and may lead to treatment discontinuation and recurrent relapses^6,7^. Prolactin increase is another common side effect in antipsychotic treatment of SCZ, with a high occurrence of 40–70% in patients^8^. It is reported that premature mortality rates are usually higher in patients suffering from SCZ relative to the general population^9^.

Previous meta-analyses have reported an increased prevalence of metabolic dysfunctions in patients with SCZ than that in the general population^10–13^, due to risk factors for metabolic abnormality including common genetic pathway, reduced physical activity, unhealthy lifestyles, sex hormones, and increased levels of stress, as well as SCZ itself^14,15^. Despite the global inconsistency among studies, marked variations in glucose, cholesterol, or triglyceride levels have been found in SCZ^13,16^. It is now accepted that glucose and lipid metabolism is impaired in patients with SCZ at the onset of disorders and before antipsychotic prescription and that some antipsychotics may worsen metabolic homeostasis in an already susceptible patient population within several weeks^17,18^. In the general population, a one-kilogram increase in body weight corresponds to a 3.1% increase in the risk of cardiovascular disease, and an 8.4% increase in the risk of type 2 diabetes^19,20^. Each mmol/L increase in triglyceride levels is associated with an increase in the risk of cardiovascular disease by 32–76%^21^.

Prolactin is a pleiotropic hormone described to be related to metabolic homeostasis, inflammatory response, and human behaviors^22^. Its specific receptors are present in abdominal fat. Prolactin impacts metabolic homeostasis by regulating critical enzymes and transporters related to glucose and lipid metabolism in several target organs^23^. Abnormalities of its different pathways have been associated with abnormal cellular proliferation and diabetes^24^.

Given that metabolic abnormalities are related to an increased risk for cardiovascular disease and that patients suffering from SCZ are increasingly predisposed to the development of metabolic dysfunction, it is therefore essential to develop and implement strategies to tackle this issue in this specific patient population. Various behavioral and pharmacological interventions have been developed to manage the conditions associated with metabolic abnormalities in SCZ. For example, the *Dance for Veterans* program aims to synergistically integrate physical components (breathing, stretching, dancing), psychological components (relaxation, creativity) and social components (group games, presenting self-developed movements to the class, synchronized movement) into a standardized and scalable program has been conducted in veterans with psychiatric disorders^25^. Another therapeutic lifestyle change (TLC) practice intervention program has also been reported to be associated with greater quality of life, more weight loss and improvements in clinical symptoms in veterans with severe mental disorders^26^. Dance/movement therapy (DMT) is an emerging therapeutic approach for improving health and well-being in the rehabilitation of patients with SCZ^27–29^. As defined by the American Dance Therapy Association, DMT therapy is the psychotherapeutic use of movement to improve well-being, mood, and quality of life^30–32^. It can be applied to individuals of all ages and is effective for individuals who experience psychological dysfunctions^28^. Additionally, DMT interventions are considered to be a very attractive and comprehensive form of exercise intervention for patients with SCZ, considering that they can regulate the biological, psychological and social needs of trainees. There is evidence that a single-DMT intervention may reduce depressive and anxiety symptoms in inpatients in psychiatric hospitals^33^. In particular, multiple DMT sessions for individuals with SCZ have revealed a reduction in negative symptoms, depression and anxiety and an increase in emotional expressivity^34,35^. Several meta-analyses also showed that DMT decreases depression and anxiety and increases quality of life and interpersonal and cognitive skills^36,37^. Our previous study has reported the efficacy of DMT on balance ability and bone mineral density in patients with SCZ^38^. However, to our best knowledge, the efficacy of multiple DMT interventions for metabolic dysfunction in long-term hospitalized veterans with SCZ has not been fully studied.

Therefore, in this study, we hypothesized that the multiple DMT interventions were effective in alleviating the metabolic dysfunction in long-term hospitalized patients with SCZ as compared to the treatment as usual. To test the hypothesis, our study would address these questions: 1) whether DMT intervention can significantly change body weight and BMI; 2) whether DMT reduced the levels of metabolic parameters in patients; and 3) whether DMT for 3 months significantly decreased the prolactin levels.

## Methods

### Patients

We performed a post hoc analysis of data from our previously published clinical study^38^. The current post hoc analyses were based on the data at baseline and at 12-week follow-up. A total of 80 hospitalized veterans with SCZ were recruited from Hebei Province Veterans Hospital in the present study. Then, eligibility screening was conducted by an experienced psychiatrist according to inclusion/exclusion criteria. The inclusion criteria included: 1) a diagnosis of SZ using the DSM-V; 2) male veterans, aged 40 to 60 years old; 3) able to understand Mandarin Chinese; 4) legally eligible to sign an informed consent form; and 6) no comorbid serious physical illness and able to cooperate with nurses to complete the activity training. The exclusion included: 1) substance dependence or abuse; 2) lower limb injury and motor dysfunction, and inability to complete DMT intervention for various reasons. These situations are often associated with the inability to complete the therapy and dropout, and could thus lead to unequal sample sizes and biased results, and threaten the validity of the results.

The study protocol was approved by the institutional review board of Hebei Province Veterans Hospital. Written informed consent was obtained from all participants. The work on patients was carried out in accordance with The Code of Ethics of the World Medical Association (Declaration of Helsinki).

Out of the 80 patients, 20 were excluded and 60 were randomly allocated to either the treatment as usual (TAU) group or the DMT plus TAU group (DMT). Two participants (1 in the TAU group and 1 in the DMT group) dropped out in the first week of treatment. Finally, 58 patients (29 patients in each group) were included in the statistical analyses. See Supplementary Fig. 1 for the recruitment and enrollment flowchart.

### Design

This was a randomized, single-blinded, controlled trial. The detailed randomization and blinding procedures were described in the Supplementary file. Eligible patients were randomly allocated (1:1) to receive either DMT intervention or TAU based on a computer-generated sequence. Raters and investigators remained blinded during the intervention.

The primary outcomes were evaluated by using a two-arm parallel group design between the TAU and DMT groups. One group member in each group was the dance movement therapist, whereas the other group members were hospitalized veterans with SCZ who chose to participate in this study.

### Interventions

A dance movement therapist who had working experience for more than 10 years in DMT intervention offered DMT for patients. Twice weekly hour-long sessions were offered over three months with a total of 24 sessions. The DMT intervention protocol was described in the Supplementary file. In brief, the therapist used a body-based approach that included movement warm-ups followed by improvisational dance and interpersonal relationships building through shared movement experiences.

The patients in the TAU group were also divided into two groups and treated as usual. TAU treatment was provided by a licensed professional and the detailed TAU procedure was described in the Supplementary file.

### Primary and secondary outcome measurements

The primary outcome was the body weight. BMI was also calculated by body weight and height a secondary outcome measure. After a 12 h overnight fast, blood samples were taken from all patients at 7:00 am. Routine metabolic biomarkers including levels of serum prolactin and lipid profiles were determined in the hospital laboratory using kits with an automatic biochemistry analyzer AU2700.

### Data analysis

Statistical analyses were conducted in SPSS 22.0. All statistical analyses were 2-sided, and the significance threshold was set at *p*-values lower than 0.05.

### Baseline analysis

The last observation carried forward (LOCF) analysis was not performed for patients who dropped out in the first week of treatment in this study. The intention to treat (ITT) analysis was used for the outcome measures. Demographic characteristics, fasting glucose levels and lipid profiles at baseline were compared between the DMT and TAU groups using chi-square tests or analysis of variance (ANOVA).

### Pre- and post-test analysis

Repeated measure analysis of variance (RM-ANOVA) was performed to compare the efficacy of DMT on metabolic parameters (the group-by-time interaction effect). There is evidence that type 1 errors can be reduced by using the RM-ANOVA. In the models, time was entered as within-effect, the treatment group was entered as between-effect, and metabolic parameters over time were dependent variables. Then, the separate repeated measure ANOVA (RM-ANOVA) tests were followed up with the tests of individual outcome variables, only if the RM-MANOVA showed a significant effect.

If individual RM-ANOVA revealed a significant interaction effect of group and time, then, the analysis of covariance with baseline value as a covariate was performed to compare the differences between the groups after treatment. If the interaction was not significant, subsequent analysis was not performed. Bonferroni correction was used to correct for multiple comparisons.

### Regression analysis

Linear regression analyses were performed to investigate the predictive role of sociodemographic and clinical characteristics and baseline metabolic biomarkers for the improvement in metabolic parameters after DMT treatment. The outcomes were defined as the changes in values between baseline and follow-up.

## Results

### Baseline comparisons

As shown in Table 1, we found that there were no significant differences in the baseline values between the DMT and TAU groups (all *p* > 0.05) (Table 1). The two groups were well-matched with respect to demographic characteristics, weight, BMI, prolactin levels, and lipid profiles at baseline. In addition, no significant difference was observed in dose of antipsychotics and disease severity between groups.

**Table 1:** Comparisons of demographic characteristics and biomarkers between the DMT group and the control group ($\bar{x}\pm s$).

Variables	TAU (*n* = 29)	DMT (*n* = 29)	t or *X*^2^ (*p*-value)*	Dropouts (*n* = 2)	Finished (*n* = 58)
Age (ys)	55.3 ± 3.9	55.4 ± 3.6	0.31 (0.97)	55.5 ± 2.12	55.4 ± 3.7
Education (ys)	8.5 ± 2.0	8.9 ± 2.3	0.87 (0.39)	8.6 ± 2.8	8.7 ± 2.2
Disease duration (ys)	34.7 ± 4.3	33.7 ± 4.9	0.75 (0.46)	33.5 ± 3.5	34.2 ± 4.6
Current disease duration (ys)	7.6 ± 4.4	8.7 ± 6.6	0.71 (0.48)	5.0 ± 1.4	8.7 ± 5.5
Dose of antipsychotics (mg/d^a^)	494.9 ± 258.3	432.1 ± 317.9	0.7 (0.42)	470.4 ± 260.3	463.5 ± 287.9
Weight (Kg)	62.5 ± 11.3	66.3 ± 9.1	1.4 (0.16)	63.9 ± 10.5	64.4 ± 10.2
BMI (Kg/m^2^)	21.9 ± 3.6	23.6 ± 3.2	0.21 (0.83)	20.2 ± 1.4	22.8 ± 3.4
Smokers *n*(%)	17 (58.6)	16 (55.2)	0.1 (0.79)	1 (50.0)	33 (56.9)
Prolactin	26.3 ± 30.2	23.6 ± 15.7	0.43 (0.67)	24.2 ± 16.7	25.0 ± 23.4
Fasting glucose	5.4 ± 0.7	5.4 ± 0.8	0.39 (0.70)	4.8 ± 0.6	5.4 ± 0.8
Triglycerides	1.6 ± 0.8	1.6 ± 0.7	0.07 (0.94)	1.1 ± 0.04	1.6 ± 0.8
Total cholesterol	4.6 ± 1.0	4.7 ± 0.9	0.23 (0.82)	3.6 ± 1.0	4.7 ± 0.9
P subscore	15.0 ± 7.8	12.9 ± 5.7	1.3 (0.25)	14.5 ± 6.8	14.0 ± 6.9
N subscore	30.7 ± 7.6	29.3 ± 10.8	0.3 (0.59)	30.0 ± 9.4	30.0 ± 9.3
G subscore	35.3 ± 9.6	34.6 ± 8.6	0.09 (0.76)	35.5 ± 9.0	34.9 ± 9.1
PANSS total score	81.0 ± 21.7	76.8 ± 22.6	0.51 (0.48)	79.5 ± 22.9	78.9 ± 22.1

^a^Antipsychotic dose was reported in chlorpromazine equivalents.

*ys* years, *BMI* Body mass index, *P* positive symptoms, *N* negative symptoms, *G* general psycho.

^*^Comparisons of baseline characteristics between the TAU and DMT groups were performed by using t-tests or chi-square showed no differences between the two groups.

No significant associations were observed between the weight or BMI and disease duration, age, fasting glucose, triglycerides, total cholesterol, and prolactin levels (all *p* > 0.05).

### DMT Intervention on BMI and metabolic markers

**Fig. 1:**
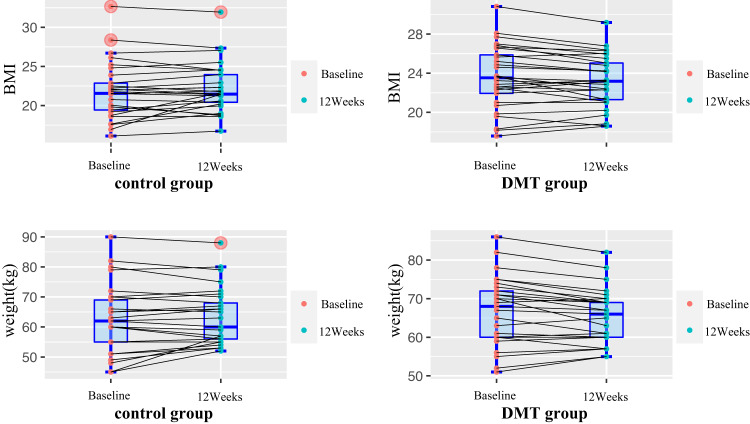
Shows the individual change from baseline to post. The outcome was on y-axis (BMI, weight etc.), time point (baseline and 12 weeks) was on x-axis in each group (DMT and TAU), with lines combining each subject-specific point across both timepoints. This provides an overview of how many subjects in each group showed improvements in the respective outcomes and how many did not.

RM-ANOVA analysis was performed to assess the efficacy of DMT intervention on the primary outcomes, including body weight and BMI. We found a significant interaction between time and treatment group on weight (F = 5.5, *p* = 0.02) and BMI (F = 5.7, *p* = 0.02) (Table 2). The average changes from baseline were 0.4 (95% CI: 0.05–0.8) for BMI and weight 1.3 (95% CI: 0.14–2.0). The individual change in weight and BMI from baseline to post is shown in Fig. 1. However, after Bonferroni correction for multiple testing (a significant *p*-value ≤ 0.05/7), the interaction effects on weight and BMI were not significant, indicating a weak effect.

**Table 2:** Comparisons of weight and lipid profiles after treatment using repeated measures ANOVA analysis ($\bar{x}$±*s*).

Variables	Control group		DMT group			
	At baseline (*n* = 29)	At follow-up (*n* = 29)	At baseline (*n* = 29)	At follow-up (*n* = 29)	Group effect F(*p*)	Group*Time interaction effect F(*p*)*
Fasting glucose	5.4 ± 0.7	5.0 ± 0.5	5.4 ± 0.8	5.2 ± 0.7	15.4 (< 0.001)^**^	2.4 (0.13)
Triglycerides	1.6 ± 0.8	1.5 ± 0.6	1.6 ± 0.7	1.4 ± 0.5	8.2 (0.006)^**^	0.5 (0.47)
Total cholesterol	4.6 ± 1.0	4.1 ± 0.8	4.7 ± 1.0	4.3 ± 0.9	29.7 (< 0.001)^**^	0.3 (0.55)
Prolactin	26.3 ± 30.2	26.2 ± 29.5	23.6 ± 15.7	26.3 ± 17.8	0.47 (0.50)	0.60 (0.44)
BMI (Kg/m^2^)	21.8 ± 3.6	22.2 ± 3.1	23.6 ± 3.2	23.3 ± 2.7	0.1 (0.74)	5.7 (0.02)^*^
Weight (Kg)	62.5 ± 11.25	63.2 ± 9.3	66.3 ± 9.1	65.1 ± 7.1	0.28 (0.60)	5.5 (0.02)^*^

^*^p < 0.05, ^**^p < 0.01.

^*^Comparisons of weight and lipid profiles before and after treatment using repeated measures ANOVA showed significant interactions on BMI and weight.

RM-ANOVA analysis was also used to assess the efficacy of the secondary outcomes, including prolactin, fasting glucose, and lipid profile. We found no significant interaction effects on fasting glucose, prolactin, triglyceride, and total cholesterol levels (all *p* > 0.05). In addition, no significant main effects of time or group on BMI and other markers of metabolic abnormalities were observed (all *p* > 0.05).

### Potential predictors of treatment response

Multiple linear regression analysis was performed to identify potential predictors of improvement in metabolic biomarkers. Since there was a significant correlation between disease duration and age, therefore, only age was added as a covariate in the models to avoid co-linearity between covariates. The results showed that disease duration of SCZ was a significant predictor for the reductions in body weight (β = −0.23, *t* = −2.4, *p* = 0.02, 95% CI: −0.43 to −0.03) and BMI (β = −0.16, *t* = −2.3, *p* = 0.03, 95% CI: −0.17 to −0.01) from baseline to week 12 in the DMT group.

## Discussion

This study found that after 3 months of treatment, body weight and BMI decreased significantly in hospitalized veterans with SCZ who received the DMT intervention compared to the TAU. In addition, disease duration was a significant predictor for the reductions in body weight and BMI after treatment. However, 3 months of DMT intervention had no effect on the levels of fasting glucose, blood lipids, and prolactin in patients.

Our study showed that 3-month DMT interventions were effective in decreasing anthropometric parameters, such as body weight and BMI in long-term hospitalized male veterans with SCZ. Our findings were in line with previous studies that the implementation of an exercise intervention regimen can result in body weight loss in the general population^39–41^. In particular, the effectiveness of group intervention programs was revealed in a systematic review by Shakeel et al. who proposed the implementation of such programs as an effective and cost-effective way to provide physical and social health benefits to residents of nursing homes^42^. Another meta-analysis by Jansen et al. supports the potential of group exercise to increase physical activity among nursing home residents^43^. Our results support that multiple DMT sessions for patients with SCZ in inpatient psychiatric hospitals may be a promising treatment option for metabolic management. In psychiatric hospitals, prolonged hospitalization, reduced outdoor activity exercise and side effects of antipsychotic medication contribute to an increased risk of metabolic abnormality in hospitalized patients with SCZ. However, it is difficult to treat metabolic dysfunction in individuals with SCZ who have been hospitalized for a long period. Music has been reported to be an important tool to support the involvement of older persons in physical exercise in nursing homes and improve their physical fitness^44–47^. DMT intervention uses music during physical activity to reduce perceived effort, depression and anxiety and improve mood. Accumulating studies have shown the positive effects of DMT on psychotic symptoms for patients with SCZ^31,48^. We speculate that this is why DMT leads to BMI and weight reductions, while other exercise treatments do not seem to be effective in patients^49^. This clinical trial provides evidence for the specific role of DMT interventions in body weight control in long-term hospitalization patients with SCZ. It should be noted that the effect size was small and conclusions should be interpreted with caution. The difference in actual change in BMI and weight (0.4 change in BMI and 1.3 kg change in weight) is very low and appears to be negligible or at the very least not clinically relevant. However, previous studies indeed reported a one-kg increase in weight leading to increased chances of cardiovascular disease and type 2 DM. Our study provides more support for the data showing that relatively nominal weight changes are associated with clinically significant changes in health factors and are clinically relevant to most patients or providers.

However, we found that the 3-month multiple DMT sessions were not effective in reducing the levels of abnormal metabolic biomarkers, such as lipid metabolism and fasting glucose, compared with the TAU group in long-term hospitalized veterans with SCZ. Our findings were consistent with the majority of studies in the general population and in patients that have shown little or no effect on metabolic parameters, such as glucose or lipid profile^50–53^. A previous review has shown that in many cases, the changes in glucose and lipid profiles were seen most in those who begin with poor health states, whose baseline health levels are farther from optimal and in most likely to improve^54^. Indeed, patients in our study were not obese or overweight (average BMI = 21.9/23.6 kg/m²) defined according to the Chinese Working Group on Obesity in China (WGOC) criteria^55^. On the other hand, our negative findings may be due to the short duration of the DMT intervention or the short post-treatment follow-up of patients. In addition, various confounding factors, such as smoking, type and dose of antipsychotic medication, can also influence BMI and metabolic parameters. Particularly, participants in the TAU group in this study were also engaged in exercise. Taken together, our pilot study supports that 3-month DMT sessions showed no effect on levels of fasting glucose and lipid profile in those inpatients with normal average BMI.

Interestingly, we found a predictive potential of disease duration for the changes in BMI and body weight after DMT therapy in this study. Consistent with previous studies, our findings suggest that there are specific subgroups of patients that benefit from exercise interventions^56–58^. Additionally, we found that those with a longer disease duration of SCZ may achieve more weight loss than those with a shorter disease duration. It is now well established that both the disease duration of SCZ and antipsychotics use are associated with increased metabolic risk^13,16^. Although the mean weight of our patients was within the normal range, previous studies have shown that patients with SCZ had higher intra-abdmonial fat, abdominal fat and waist to hip ratios compared to healthy controls, despite no significant differences in weight between patients and controls^59^. Prospective longitudinal studies have also found that long-term antipsychotic treatment can modulate the metabolic system and increase abdominal fat, body weight and BMI^16^. We speculate that patients with shorter disease duration imply a shorter duration of antipsychotic medication and may suffer less from structural and functional deficiency to adipose tissue and associated metabolic system, whereas patients with longer disease duration may have relatively worse adipose tissue function and lipid metabolism and therefore more improvement can be achieved with DMT interventions in this specific subgroup of patients.

In our study, the discontinuation rate is 3.3% (2/60), which is lower than the recommended value for psychosocial research in patients with SCZ^60^, and lower than what has been reported in other studies on exercise intervention^61–63^. Although adherence to exercise intervention regimen is particularly important for the long-term care of patients with SCZ^64^, discontinuation rates of exercise interventions pose an important challenge in SCZ, and rates vary substantially across studies^61–63^. The low discontinuation rate in this clinical trial suggests that long-term hospitalized patients with SCZ have good treatment adherence to DMT interventions and their suitability for implementation in psychiatric hospitals. The DMT intervention is known to use rhythmic movement as an organizing force and a cohesive group process to form group psychotherapy. It creates a safe environment for patients to express themselves authentically and develop a sense of acceptance. As a result, long-term inpatients showed low dropout rates in this study.

There were several limitations in this study. First, this was a pilot study with a small sample size for a clinical trial to examine the efficacy of a DMT intervention on metabolic parameters in patients with SZ and therefore was not aimed to produce generalizable results. In addition, the comparatively small sample size may lead to positive or negative results due to the lack of power. Hence, our findings need to be replicated in a prospective study with larger sample sizes. Second, the duration of the DMT intervention is short, and we were unable to investigate long-term improvements in metabolic markers in veterans. Notably, some patients recruited in the present study expressed a preference for long-term treatment. Further studies are needed to investigate the efficacy of DMT interventions for more than three months, e.g., fifty-two weeks^27^. Third, the sample is exclusively male in this study, which limited the generalization of our findings. Further investigations should be performed to assess whether DMT interventions show efficacy in female patients with SCZ. Fourth, participants in this study were not overweight (BMI = 22). The changes in weight/BMI may not be the most clinically significant outcome measures. As mentioned in the discussion, waist to hip ratio may be more relevant in patients whose weight was in the normal range. However, we did not examine it in this study, which was warranted to be measured in the future study. Fifth, previous studies have shown that general exercise treatments are not effective in reducing BMI and weight reduction in patients with Sch, as reported in participants in the control group engaged in exercise in our study. It would be interesting to conduct a head-to-head trial of an exercise program versus dance movement therapy with a large sample size in a future study to evaluate the unique benefits of dance therapy. Sixth, our study focused on BMI and weight reduction after treatment with DMT. We assessed only baseline clinical symptoms and did not assess the psychotic symptoms at follow-up. It would be very interesting to assess PANSS at follow-up and investigate if improvements on the physical level are directly related to improvements in the clinical outcomes (symptoms, cognition, and functioning), especially in patients who were not obese or overweight. Thus, future studies should add an analysis (e.g. logistic regression) in which they examine if patients who show reductions in BMI/weight are also more likely to show improvements in symptoms, cognition, functioning, quality of life, etc.

## Conclusions

Metabolic abnormalities are one of the serious side effects in patients with SCZ. Long-term hospitalized patients with SCZ are at increased risk of obesity and other metabolic parameters due to lack of exercise and antipsychotic medication use. Our 12-week DMT program reduced anthropometric measures such as body weight and BMI in patients with SCZ. The findings of our study contribute to the introduction of valuable multiple DMT sessions in psychiatric hospitals and provide a validated intervention strategy in clinical practice for weight loss in long-term hospitalized veterans with SCZ. Further research will help to develop important recommendations for action and guidance to promote the health and well-being of patients with SCZ.

### Supplementary information

Supplementary file

Supplementary Figure

## Data Availability

The datasets generated and analyzed during the current study are available from the corresponding author upon reasonable request.
