# Supplementary material for: Dance/movement therapy for improving metabolic parameters in long-term veterans with schizophrenia
Source: Schizophrenia (Heidelb). 2024 Feb 22;10(1):23. doi: 10.1038/s41537-024-00435-7 (PMC10884034; doi:10.1038/s41537-024-00435-7)
Supplement: Supplementary file 1 — Supplementary file [file 41537_2024_435_MOESM1_ESM.docx]

***Supplementary File 1***

**S1. *Randomization and Masking***

This was a randomized, single-blinded, controlled trial. There was no blinding due to the experience of therapists in applying the intervention. Outcome evaluators and patients remained blinded during the 12 weeks of treatment, however, patients sometimes shared their intervention experience, which may lead to the break of blinding. Treatment allocation was conducted using a standard equal random assignment method. Eligible patients were randomly allocated (1:1) to receive either DMT intervention or TAU based on a computer-generated sequence. The independent third party who was blinded to the subject groups divided the patients into two groups. The trial statisticians (XL and HG) were masked from intervention allocation. Measurement of metabolic parameters was completed by XL and XZ who were blinded to group allocation and instructed not to ask about participant’s intervention allocation.

***S2. Detailed treatment protocol for DMT and TAU.***

***DMT protocol***

The therapist used a body-based approach that included movement warm-ups followed by improvisational dance, the narration of the process by the patients and the therapist, and interpersonal relationships building through shared movement experiences. First session: meeting, warming up, determining the group rules, explaining the principles, and determining the expectations activities. Subsequent sessions: the greeting is completed with warm-up, initiation, continuation, and closing activities. During the warm-up phase, the whole group comes together to form a circle, generally standing, in order to ensure the group dynamic. It starts with simple warm-up exercises such as breathing and muscle relaxation and continues with body awareness exercises. Of note, to ensure the proper delivery of the protocol and the proper time for interpersonal connection with each group member, we divided the patients into 2 groups (15 and 14 patients each) on Mondays and Wednesdays or on Tuesdays and Thursdays.

***TAU protocol***

The patients in the control group did not participate in the DMT intervention but were treated as usual. The TAU group sessions were conducted by licensed professionals employed at the hospital. The patients received regular daily antipsychotic medication and supportive psychotherapy once a week. Supportive psychotherapy was applied to the treatment of the patients once a week as a total of 12 sessions of 45 minutes over three months. Patients were divided into two groups of 15 and 14 each. They also participated in daily activities from Monday to Friday for at least two hours each day, including indoor activities (watching TV, playing games, playing poker) and outdoor activities (walking and doing radio gymnastics).
