# Supplementary material for: Dance/movement therapy for improving metabolic parameters in long-term veterans with schizophrenia
Source: Schizophrenia (Heidelb). 2024 Feb 22;10(1):23. doi: 10.1038/s41537-024-00435-7 (PMC10884034; doi:10.1038/s41537-024-00435-7)
Supplement: Supplementary file 2 — Supplementary Figure [file 41537_2024_435_MOESM2_ESM.ppt]

## Slide 1
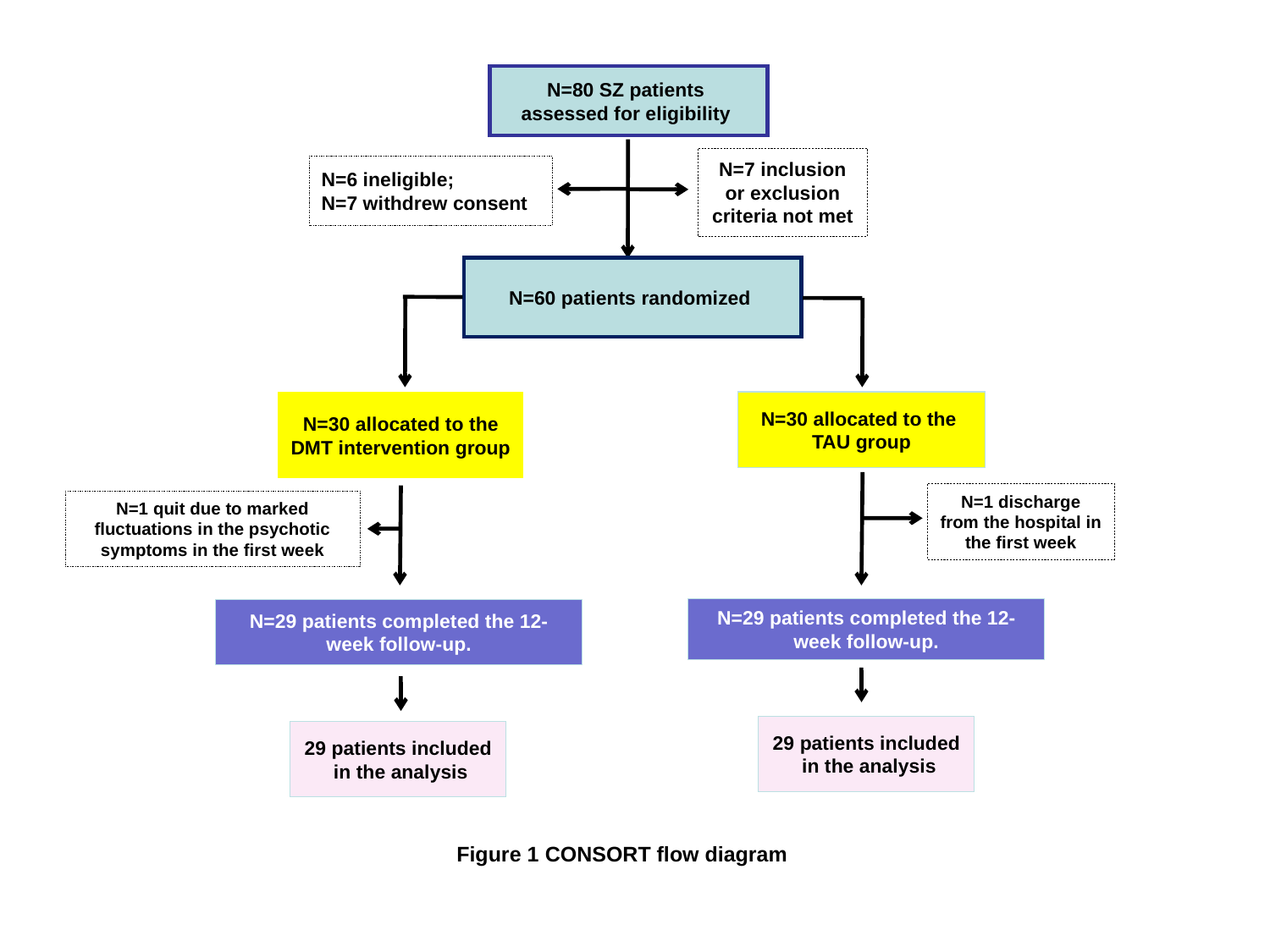

N=80 SZ patients assessed for eligibility
N=7 inclusion or exclusion criteria not met
N=6 ineligible;
N=7 withdrew consent
N=60 patients randomized
N=30 allocated to the DMT intervention group
N=30 allocated to the
TAU group
N=1 discharge from the hospital in the first week
N=1 quit due to marked fluctuations in the psychotic symptoms in the first week
N=29 patients completed the 12-week follow-up.
N=29 patients completed the 12-week follow-up.
29 patients included
 in the analysis
29 patients included
 in the analysis
Figure 1 CONSORT flow diagram
